# Human biomarkers associated with low concentrations of arsenic (As) and lead (Pb) in groundwater in agricultural areas of Thailand

**DOI:** 10.1038/s41598-021-93337-y

**Published:** 2021-07-06

**Authors:** Pokkate Wongsasuluk, Srilert Chotpantarat, Wattasit Siriwong, Mark Robson

**Affiliations:** 1 International Postgraduate Programs in Environmental Management, Graduate School, Chulalongkorn University, Bangkok, 10330 Thailand; 2 Center of Excellence on Hazardous Substance Management (HSM), Chulalongkorn University, Bangkok, 10330 Thailand; 3 College of Public Health Sciences, Chulalongkorn University, Bangkok, 10330 Thailand; 4 Department of Geology, Faculty of Science, Chulalongkorn University, Bangkok, 10330 Thailand; 5 Research Program on Controls of Hazardous Contaminants in Raw Water Resources for Water Scarcity Resilience, Center of Excellence on Hazardous Substance Management (HSM), Chulalongkorn University, Bangkok, 10330 Thailand; 6 Research Unit Control of Emerging Micropollutants in Environment, Chulalongkorn University, Bangkok, Thailand; 7 Thai Fogarty ITREOH Center, Chulalongkorn University, Bangkok, 10330 Thailand; 8 New Jersey Agricultural Experiment Station, Rutgers University, New Brunswick, NJ USA; 9 School of Environmental and Biological Sciences, Rutgers University, New Brunswick, NJ USA

**Keywords:** Environmental impact, Environmental sciences, Hydrology, Biomarkers

## Abstract

Human biomarkers were used to evaluate the lead (Pb) and arsenic (As) exposure of local people who lived in an agricultural area with intense agrochemical usage and who consumed groundwater. Although the heavy metals/metalloids in the groundwater were at low concentrations, they could cause adverse effects due to a high daily water intake rate over the long term. Biomarkers (hair, fingernails and urine) were collected from 100 subjects along with the local shallow groundwater and tap water, which is the treated deep groundwater, and investigated for the concentrations of As and Pb. Shallow groundwater had an average pH of 5.21 ± 1.90, ranging from 3.77 to 8.34, with average concentrations of As and Pb of 1.311 µg/L and 6.882 µg/L, respectively. Tap water had an average pH of 5.24 ± 1.63, ranging from 3.86 to 8.89, with the average concentrations of As and Pb of 0.77 µg/L and 0.004 µg/L, respectively. The levels of both As and Pb in the hair, fingernails and urine of shallow groundwater-consuming residents were greater than those in the hair, fingernails and urine of tap water-consuming residents. Interestingly, the As level in urine showed a linear relationship with the As concentration in groundwater (R^2^ = 0.91). The average water consumption rate was approximately two-fold higher than the standard; thus, its consumption posed a health risk even at the low As and Pb levels in the groundwater. The hazard index (HI) ranged from 0.01 to 16.34 (average of 1.20 ± 2.50), which was higher than the acceptable level. Finally, the concomitant factors for As and Pb in the urine, hair and nails from both binary logistic regression and odds ratio (OR) analysis indicated that groundwater consumption was the major concomitant risk factor. This study suggested that direct consumption of this groundwater should be avoided and that the groundwater should be treated, especially before consumption. In conclusion, urine is suggested to be a biomarker of daily exposure to As and Pb, while for long-term exposure to these metals, fingernails are suggested as a better biomarker than hair.

## Introduction

Heavy metal/metalloid contamination in freshwater supplies, especially groundwater, is a major concern worldwide^1–8^. Heavy metals/metalloids have high toxicity, even at low concentrations, and can persist in groundwater for a long time. Local people in many countries still use groundwater as their major water supply and thus, could be exposed to heavy metals by consuming this water. Risk assessment can be used to predict health effects to prevent health risks from high exposure to heavy metals/metalloids. Heavy metals mostly found in environment such as arsenic (As), lead (Pb), copper (Cu), cadmium (Cd), zinc (Zn), etc.^9–11^. These heavy metals can be accumulated through blood circulation system in human body after exposure and can cause adverse health effects. There are three routes of heavy metals exposure, which are ingestion, dermal, and inhalation. The primary exposure pathway of As is ingestion through water and food, while inhalation and dermal absorption are considered as minor pathway^12^.

In addition, biomarkers can be used as indicators to measure exposure levels^3,13–20^. The mechanism of human body can prevent the heavy metals high accumulation with many processes such as, excretion through urine, feces, sweat, breast milk, hairs, nail etc. Urine is a major route to excrete heavy metals out of human body^16,21^. As and Pb can cause adverse health effects to their target organ, such as lung, kidney, liver, brain, immunological, cardiovascular etc. The adverse health effects from As and Pb exposure are generally classified into two types which are acute effects and chronic effects^3^. The chronic exposure to As through contaminated water may cause for various health intimidations. Arsenic increases oxidative stress, upregulates proinflammatory cytokines and inflammatory mediators, and induce cardiovascular abnormalities. Moreover, As is carcinogen that can induced carcinogenicity then cause cancer in human. Ingestion or oral exposure can affect chronic toxicity that can produce skin lesions or hyperpigmentation^12,22^.

Biomarkers can be used to determine exposure to potentially toxic heavy metals/metalloids. Compared to biomarkers such as blood or internal organ tissues, which require invasive sampling, noninvasive biomarkers, such as urine, hair and nails, seem to be easier-to-access indicators of exposure. Moreover, urine is the main route of excretion from recently ingested contaminated food and water^15,16^.

Most research has focused on highly contaminated areas, such as mining or industrial areas with heavy metal-contaminated groundwater^4,5,23–27^, and on using human biomarkers in high-exposure circumstances. In contrast, this study focused on low-contamination sites, such as an area with intense agricultural activity and long-term agrochemical use^28–31^. Some groundwater wells in this study had heavy metal concentrations at the low contamination levels, but biomarkers revealed that the heavy metals had accumulated in the bodies of local residents from long-term exposure via water intake at a high rate. Many studies in India have reported that the major sources of heavy metals released to water are heavy metal-containing fertilizers and pesticides in agricultural fields^32–35^. Similarly, a study on heavy metal contamination in the Yamuna River, India, found that agricultural runoff carrying fertilizers containing heavy metals caused contamination by heavy metals in the river. The abundances in the heavy metals in the river were as follows: iron (Fe) > copper (Cu) > zinc (Zn) > nickel (Ni) > chromium (Cr) > lead (Pb) > cadmium (Cd)^36^. A study in Bangladesh by^37^ found that arsenic (As) in urine was well correlated with As in drinking water. The most significant linear relationship was shown between the concentration of As in drinking water (range < 0.5–332 µg/L) and that of As in urine. In addition to As exposure via drinking water, seafood ingestion is also a source of As^37^. In terms of human biomarkers, urine is the most frequently used and is important for investigating the associated factors that may contribute to As in urine^38^. Moreover, the heavy metals in drinking water were related to heavy metals in human biomarkers, consisting of urine and hair from local people in the villages in the Red River Delta, Vietnam. This research identified high levels of As in water (≥ 50 μg/L As), and 64% of the hair samples contained As concentrations greater than 1 μg/g^39^. Furthermore, several studies have reported adverse health effects, both carcinogenic and noncarcinogenic, from heavy metal exposure. Long-term exposure to heavy metals may cause cancer in internal organs such as the kidney, lung, and liver. In addition, health effects from heavy metals in drinking water included skin damage, respiratory disorders, gastrointestinal disorder, renal failure, anemia, cardiovascular diseases, and hypertension^40–42^.

The study area is located in a cultivated farming area in Ubon Ratchathani Province, located in the northeastern region of Thailand. Large amounts of agrochemicals (pesticides and fertilizers) are typically used in this area. The pesticides used are mainly organophosphates, such as chlorpyrifos and profenofos, while the fertilizers are largely ammonium nitrate fertilizers^10^. Pesticides and fertilizers increase the acidity of soil and shallow groundwater, where decreasing groundwater pH is positively correlated with increasing nitrate concentrations in groundwater. The increasing groundwater acidity causes the soil-bound heavy metals/metalloids to be more easily released (leached) into the groundwater. Moreover, contamination by several heavy metals, such as chromium (Cr), nickel (Ni), copper (Cu), zinc, arsenic (As), cadmium (Cd), mercury (Hg) and lead (Pb), was found in agricultural areas that had used fertilizers^3^. In addition^43^, found heavy metals, including As, Cu, Pb and Zn, in soil samples from this agricultural study area. The As, Cu, Pb, and Zn concentrations in the soils ranged from 0.07 to 0.33 mg/kg, 4.39 to 11.75 mg/kg, 0.11 to 0.28 mg/kg, and 70.66 to 110.20 mg/kg, respectively^3,43^. The soil textures in this agricultural area consisted of 3 types: sandy loam, loamy sand and sand, which possibly released some soil-bound heavy metals/metalloids to groundwater. Furthermore, Wongsasuluk et al.^45^ found that the pH of groundwater was acidic, and some groundwater wells were found to have As concentrations higher than the groundwater drinking standard.

Three types of human biomarkers were used in this study: urine, hair and fingernails. Urine was used to assess daily exposure^15,16^, while hair and fingernails were applied as biomarkers to measure long-term exposure^3,17–20,46–48^. This study focused on the concentrations of the heavy metal Pb and the metalloid As in the rainy season, as they both have high toxicity and can result in many detrimental health effects to humans even when present in low concentrations. Health risk assessment and biomarker data were evaluated to compare shallow groundwater-consuming (SGWC) and tap water-consuming (TWC) local people in the study area. The aims of this study were to (i) measure the As and Pb levels in groundwater and tap water in this intensive agricultural area, (ii) determine the concentrations of As and Pb in three biomarkers (urine, hair and fingernails) in SGWC and TWC residents, and (iii) explore the concomitant factors of heavy metals/metalloids in these subjects with these biomarkers.

## Materials and methods

### Study area

**Figure 1:**
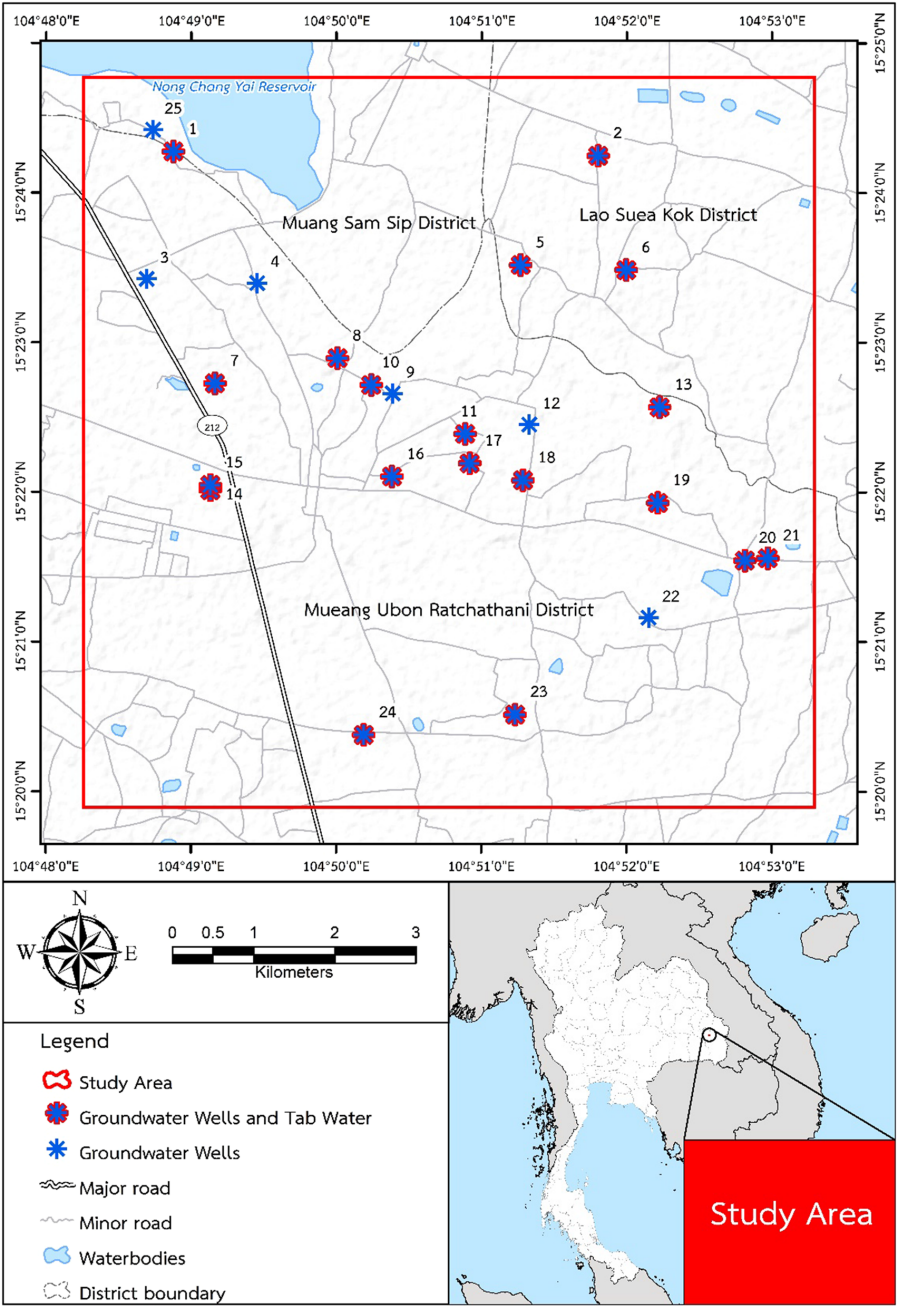
Study area location in Ubon Ratchathani, Thailand, showing the 25 groundwater well stations and those where residents has access to tap water as well (adapted from^34^, Springer Nature, Environmental Geochemistry and Health, by Wongsasuluk et al. Copyright 2017).

The study area was located in Ubon Ratchathani Province, northeastern Thailand, within the UTM range of East 1,695,000–1,704,000 and North 479,000–469,000 (latitude 15° 13′ 44″ N or 15.228889, longitude 104° 51′ 15″ E or 104.854167, Zone 47P, Datum WGS 84) (Fig. 1).

This area has been constantly and thoroughly cultivated long term with heavy agrochemical usage to grow mainly chilis and rice. The local shallow groundwater wells were generally located in agricultural areas close to residents. The shallow groundwater was pumped up from small local wells, which have been used for > 10 years. The tap water samples in this study area were collected from faucets located close to participating residences. The raw tap water came from deep groundwater and not the sampled shallow groundwater wells, which the local people pump individually. Tap water generally comes from a deep aquifer, which is deeper than 40 m and managed by the local government. The filtration of tap water was conducted by the local government or village headman. The tap water was cleaned in a pressurized sand filter tank with a diameter of 1.15 m and height of 1.20 m. The layer sequence of the filter materials in the filter tank was as follows: glass sand or silica sand (grain size, 0.125–0.25 mm), 240 L; coke, 40 L; fine sand (grain size, 0.5–1.0 mm), 120 L; sand (grain size, 1.0–2.0 mm), 80 L; fine gravel (grain size, 4.0–8.0 mm), 80 L; small gravel (grain size, 8.0–16.0 mm), 80 L; and gravel (grain size, 16–30 mm), 160 L.

### Sampling and analytical methods

This research was approved by the Ethics Review Committee for Research-Involving Human Research subjects—Health Science Group, Chulalongkorn University (COA. 002/2558), which provided certification documents. The methods and questionnaire met all applicable standards with regard to the Ethics Review Committee. According to the concerned field, the research was carried out with full accountability following due ethical procedures. Furthermore, informed consent was obtained from all participants (100 volunteer residents) who permanently lived in agricultural areas. Participants were local males and females older than 18 who volunteered to be subjects.

Participant information was obtained via face-to-face interviews. Urine, hair and fingernail samples were collected along with the water sources that the participants generally drank. Hair and nail samples were washed with acetone as reported previously^3,45^, and then microwave digestion was used to digest the hair and nails before analysis by inductively coupled plasma mass spectrometry (ICP-MS) to determine the concentrations of As and Pb. For urine, As and Pb were detected using atomic absorption spectrometry (AAS)^45^. Shallow groundwater and tap water samples were first preserved with the addition of nitric acid, and then the concentrations of As and Pb were measured by ICP-MS. For microwave digestion, the method followed the Milestone Microwave Laboratory System, 1998. Inductively coupled plasma spectrometry-mass spectrometry (ICP-MS) (Perkin-Elmer SCIEX, ELAN 6000) was performed following the AWWA (American Water Works Association) in-house method TE-CH-038, which is based on Standard Methods for the Examination of Water and Wastewater by the AWWA (22nd edition, 2012, part 3030E). Based on the standard of the American Conference of Governmental Industrial Hygienists (ACGIH) process, atomic absorption spectrophotometry (AAS) was carried out to analyze the amounts of heavy metals^3^. For the determination of As and Pb in water, hair, and nail samples by ICP-MS, a standard curve for As and Pb was generated. The standard curves were created from eight concentration points ranging from 0.5 to 100 µg/L, with r-squared values of 0.9998 and 0.9999 for As and Pb, respectively. In the analysis of all heavy metals, deionized water (DI water) was used as a 0.000 µg/L blank. For all heavy metals, the ICP-MS detection limits were 0.001 µg/L, the LODs were calculated as SD*3, and the SD was 0.000 µg/L. For As, the standard curve was plotted from 5 concentration points ranging from 20 to 200 µg/L, with an r-squared value of 0.9987. The standard curve for Pb ranged from 5 to 80 µg/L, and the r-squared was 0.9993. Dilute (1%) nitric acid was used as a 0.00 µg/L standard blank. The details of the QA/QC and analytical performance were used to validate the analysis method for very low heavy metal concentrations in groundwater, and the % relative accuracy was calculated from 10 ICP-MS measurements per heavy metal compared with a CRM, as shown in the Supplementary Information (Tables S1 and S2). The certified reference material (CRM) was a standard solution **(**Category A: Chemical composition) of As, Cd, Pb diluted to 10 ppb and Hg diluted to 5 ppb.

### Data analysis

The personal information of the 100 participants received from interviews was analyzed and used in a health risk assessment. Both the carcinogenic risk and noncarcinogenic risk of As and Pb were investigated based on the four steps of risk assessment: (i) hazard identification (HI), (2) dose–response assessment, (3) exposure assessment and (4) risk characterization^3^.

The dependent variables were expressed as As and Pb in the three biomarker samples. Concomitant factors, such as sociodemographic, personal, exposure, and environmental factors, were defined as independent variables.

The Mann–Whitney U-test (2-tailed) was used to classify significant differences between the SGWC and TWC residents. Kendall and Spearman tests were used to show the relationships between the concentrations of As and Pb in all three biomarkers and the consumed water. Binary logistic regression and odds ratios (ORs) were used to evaluate the concomitant risk factors related to As and Pb. The formula shown in Eq. (1) was used to predict the *logit* of the probability of the presence of the characteristic of interest:

1 $$\log it\left( p \right) = {\text{b}}_{0} + {\text{b}}_{1} {\text{X}}_{1} + {\text{b}}_{2} {\text{X}}_{2} + {\text{b}}_{3} {\text{X}}_{3} + \cdots + {\text{b}}_{{\text{k}}} {\text{X}}_{{\text{k}}}$$

where *p* is the probability of the presence of the characteristic of interest. The logit transformation is defined as the logged odds and is shown in Eq. (2):

2 $${\text{ODDs}} = \left( {\frac{p}{{1 - p}}} \right)\;{\text{and}}\;\log it\left( p \right)~ = \ln \left( {\frac{p}{{1 - p}}} \right)$$

## Results and discussion

### Characteristics of the study area

#### Characteristics of the groundwater wells

The average water level was 126.9 ± 14.0 m above mean sea level (amsl) with the average depth from ground surface to the groundwater level of 2.4 ± 1.7 m, respectively. The average pH of the shallow and tap water (treated deep groundwater) were 5.21 ± 1.90 and 5.24 ± 1.63, respectively. The average electrical conductivity (EC) of the shallow and tap water (treated deep groundwater) were 242.90 µS/cm (range of 98– 382.5 µS/cm) and 339.59 µS/cm (range of 42.1–1223.0 µS/cm), respectively, while the oxidation reduction potential (ORP) were 408.58 µS/cm (range of 328.7– 453.4 mV) and 345.32 mV (range of 165–509.2 mV), respectively. The characteristics of the groundwater wells are summarized in Table 1.

**Table 1:** Characteristics of the water samples.

Locations		Well St	pH	EC (µS/cm)	ORP (mV)	Depth to water level (m)	Groundwater level (m, amsl.)	As in GW (µg/L)	Pb in GW (µg/L)	As in Tap (µg/L)	Pb in Tap (µg/L)
N	E										
15° 24′ 16.5″ N	104° 48′ 52.6″ E	1	4.35	1200.0	261.4	2.5	130.5	6.18	0.02	1.02	nd.*
15° 24′ 14.9″ N	104° 51′ 48.1″ E	2	6.72	337.0	235.3	2.1	136.9	1.30	0.11	0.89	nd.*
15° 23′ 25.4″ N	104° 48′ 41.6″ E	3	8.34	98.0	427.5	1.9	128.1	0.02	11.02		
15° 23′ 23.7″ N	104° 49′ 27.3″ E	4	4.16	296.0	391.0	2.2	129.8	0.01	5.73		
15° 23′ 31.1″ N	104° 51′ 16.1″ E	5	7.00	322.5	301.1	1.9	127.1	1.29	nd.*	nd.*	nd.*
15° 23′ 29.1″ N	104° 51′ 59.8″ E	6	7.26	73.9	289.2	3.1	136.9	1.08	nd.*	1.27	nd.*
15° 22′ 43.5″ N	104° 49′ 9.8″ E	7	3.86	159.0	509.2	1.2	131.8	0.13	15.79	1.17	nd.*
15° 22′ 53.7″ N	104° 50′ 0.5″ E	8	4.19	192.4	436.1	2.1	84.9	0.25	13.12	1.63	nd.*
15° 22′ 39.6″ N	104° 50′ 23.3″ E	9	3.77	382.5	453.4	2.0	128.0	0.34	37.11		
15° 22′ 43.0″ N	104° 50′ 14.4″ E	10	4.05	192.8	406.8	0.6	126.4	0.15	21.31	0.07	nd.*
15° 22′ 23.4″ N	104° 50′ 53.3″ E	11	5.25	161.1	280.8	6.0	121.0	0.09	0.53	nd.*	nd.*
15° 22′ 27.2″ N	104° 51′ 19.8″ E	12	4.04	197.2	438.2	2.3	117.7	nd.*	18.17		
15° 22′ 34.1″ N	104° 52′ 13.7″ E	13	8.89	983.0	165.0	0.9	128.1	4.18	0.38	1.60	nd.*
15° 22′ 0.7″ N	104° 49′ 8.0″ E	14	7.18	306.0	351.8	1.8	134.3	2.79	nd.*	1.66	nd.*
15° 22′ 2.8″ N	104° 49′ 8.0″ E	15	4.00	42.1	374.4	3.1	116.9	0.30	0.05	1.46	nd.*
15° 22′ 6.3″ N	104° 50′ 23.0″ E	16	4.38	63.3	353.0	1.9	145.1	nd.*	2.52	1.47	nd.*
15° 22′ 11.5″ N	104° 50′ 55.1″ E	17	3.88	232.5	341.2	1.3	122.7	1.17	nd.*	0.24	nd.*
15° 22′ 4.8″ N	104° 51′ 17.2″ E	18	4.13	332.0	328.7	2.4	138.7	0.84	nd.*		
15° 21′ 55.7″ N	104° 52′ 12.9″ E	19	4.17	149.8	441.9	2.2	129.8	0.20	33.17	nd.*	nd.*
15° 21′ 32.7″ N	104° 52′ 48.9″ E	20	4.06	156.6	450.3	4.0	128.0	nd.*	13.02	nd.*	nd.*
15° 21′ 33.6″ N	104° 52′ 58.4″ E	21	4.27	66.3	352.0	1.0	139.1	nd.*	nd.*	0.55	nd.*
15° 21′ 9.8″ N	104° 52′ 9.2″ E	22	4.11	153.4	460.4	1.3	132.7	nd.*	nd.*	nd.*	nd.*
15° 20′ 31.0″ N	104° 51′ 14.0″ E	23	4.17	437.5	276.9	1.9	142.1	0.99	nd.*	0.76	nd.*
15° 20′ 22.8″ N	104° 50′ 11.5″ E	24	7.75	1223.0	274.3	8.0	90.0	8.87	nd.*	nd.*	0.07
15° 24′ 25.2″ N	104° 48′ 44.3″ E	25^a^	6.83	151.7	412.7	0.0	140.0	2.63	nd.*		
		Ave	5.16	316.4	360.5	2.4	126.9	1.31	6.88	0.77	0.004
		SD	1.66	328.1	84.7	1.7	14.0	2.18	10.86	0.66	0.02
		Min	3.77	41.9	164.4	0.0	84.9	nd.*	nd.*	nd.*	nd.*
		Max	8.89	1223.0	509.9	8.0	145.1	8.87	37.11	1.66	0.07
		Med						0.30	0.11	0.83	nd.*
		LOD						0.001	0.001	0.001	0.001

*nd: no detection or lower than the limit of detection.

**Station had no tap water. *LOD* limit of detection.

^a^Station no.25 is surface water at reservoir.

#### Concentrations of As and Pb in shallow groundwater wells and tap water

The average As and Pb levels in the groundwater were 1.31 ± 2.18 µg/L and 6.88 ± 10.86 µg/L, respectively, whereas the average As and Pb levels in tap water were 0.77 ± 0.66 µg/L and 0.004 ± 0.017 µg/L, respectively (Table 1). In addition, nine out of 25 shallow groundwater wells (stations 3, 7–10, 12, 16, 19 and 20) had Pb concentrations above the Thailand groundwater standard for consumption, which is 10 ppb for both As and Pb^49,50^. For the results of Mann–Whitney U-test, the comparison between As concentration in groundwater and tap water is not significant while Pb is significant different (p < 0.05) between groundwater and tap water. Comparison of the As and Pb concentrations with a previous study in the dry season^3^ revealed that the amounts of Pb and As in the shallow groundwater were slightly lower during the rainy season (this study) than in the dry season by 1.58 ± 0.03 and 6.90 ± 0.08 µg/L for As and Pb, respectively. The rainwater might percolate into the shallow groundwater and dilute the As and Pb concentrations.

### Human health risk assessment

The 100 local people selected in this study had an average weight of 59.9 ± 12.8 kg, average height of 157.6 ± 7.31 cm and average age excluding children (all 100 participants were older than 18 years) of 45.8 ± 13.8 years of age. Interestingly, the daily water consumption rate of the participants in this study area was very high, at an average of 4.21 ± 2.73 L/day (minimum and maximum of 1.25 and 12.5 L/day, respectively). The maximum amount of drinking water consumed per day was 12.5 L/day, because the study area is located in a tropical zone. The temperature during daytime was higher than 40 degrees Celsius. Most of the participants were agriculturalists (78%). To produce a large amount of agricultural products, therefore, they had long working hours (approximately 10 h/day) of farming in the sunshine in a hot climate, leading to a high water consumption rate^3^. This is the reason why they have a high water drinking rate. In addition, based on a previous study in this area, the groundwater consumption by local people was quite high, at 3.6 ± 2.1 L/day/person for adults and 2.6 ± 1.0 L/day/person for the elderly^51^. Regarding food exposure, the personal information from the questionnaires included dietary seafood consumption, which is major source of heavy metals, but the result showed no seafood consumption by the participants. The location of this province is very far from a sea and seafood is expensive, and most subjects were agriculturalists with low income. Therefore, they generally consumed groundwater and were not exposed to heavy metals via seafood. Arsenic in rice can be a source of exposure, but rice may be not a major concern comparing with drinking water or seafood. Since As in rice appear to be not mainly accumulate in rice gain, but store in other parts which people do not consume. The root of rice is able to absorb and accumulate large amounts of As, but only small amounts are translocated to the rice grain and tillers. Arsenic concentrations in rice tissues decrease from the root to the grain. Some rice varieties have been developed that are resistant to high soil As concentrations and are not able to translocate the metalloid toward the root. Arsenic shared maximum As load in rice straw while it is considerably low in grains^52,53^.

There were 58 subjects who generally drink shallow groundwater and 42 subjects who drink tap water. Regarding the As levels in the SGWC group, 11 of 58 groundwater-consuming participants (19.0%) were at risk of experiencing noncarcinogenic health effects, and 10 of 58 participants (17.2%) were at risk of developing cancer. Regarding the Pb levels in the SGWC group, 21 of 58 groundwater-consuming participants (36.2%) had noncarcinogenic risk. However, there was no health risk for the TWC participants. For As in groundwater, the average cancer risk was 5.30E-07 (range of 0–7.71E-06), while in the TWC group, the average cancer risk was 2.95E-08 (range of 3.15E-07 to 5.76E-08).

For the noncarcinogenic risk, the hazard quotient (HQ) of As was 0.63 (range of 0.01–8.26) in the SGWC group and 0.04 (range of 0.01–0.26) in the TWC group. For the consumed water, the Pb-associated HQ was an average of 1.41 (range of 0.01–16.32) in the SGWC group and 0.01 (range of 0.001–0.01) in the TWC group, while the HI for the consumed water of 100 participants related was an average of 1.20 ± 2.50 (range of 0.01–16.34), greater than the acceptable level. The human health risk assessment for the As and Pb at each well for the groundwater-consuming participants is reported in Table 2.

**Table 2:** Health risk assessment for the As and Pb levels in drinking water.

Groundwater for consumption					Tap water for consumption			
Station	As-Cancer risk	As-HQ	Pb-HQ	HI	Station	As-Cancer risk	As-HQ	Pb-HQ
Well 3	4.24E−11	< 0.01	1.13	1.13	Well 1	6.64E−08	0.12	< 0.01
Well 4	7.81E−13	< 0.01	0.14	0.14	Well 2	4.75E−08	0.05	< 0.01
Well 7	1.03E−09	< 0.01	1.40	1.40	Well 4	7.75E−16	0.01	< 0.01
Well 8	4.39E−09	< 0.01	1.53	1.54	well 5	9.24E−16	0.01	< 0.01
Well 9	9.08E−09	0.01	10.12	10.13	Well 6	1.18E−07	0.15	< 0.01
Well11	4.53E−10	< 0.01	< 0.01	< 0.01	Well 9	5.70E−16	< 0.01	< 0.01
Well 12	0.00E + 00	< 0.01	2.33	2.33	Well 10	2.56E−10	< 0.01	< 0.01
Well 13	1.29E−06	1.71	< 0.01	1.71	Well 11	1.46E−15	< 0.01	< 0.01
Well 14	9.91E−07	1.25	< 0.01	1.25	Well 17	5.20E−09	< 0.01	< 0.01
Well 15	4.19E−09	< 0.01	< 0.01	< 0.01	Well 18	5.64E−16	< 0.01	< 0.01
Well16	0.00E + 00	< 0.01	0.03	0.03	Well 19	7.79E−08	0.07	< 0.01
Well 18	4.73E−08	0.07	< 0.01	0.07	Well 20	1.09E−15	< 0.01	< 0.01
Well 19	1.90E−09	< 0.01	5.02	5.02	Well 21	3.61E−08	0.02	< 0.01
Well 20	0.00E + 00	< 0.01	1.33	1.33	Well 22	2.78E−16	< 0.01	< 0.01
Well 21	0.00E + 00	< 0.01	< 0.01	< 0.01	Well 23	1.30E−08	0.04	< 0.01
Well 22	0.00E + 00	< 0.01	< 0.01	< 0.01				
Well 23	7.36E−08	0.08	< 0.01	0.08				
Well 24	4.27E−06	4.92	< 0.01	4.92				
Avg. ± SD	5.30E−07 ± 1.42E−06	0.63 ± 1.53	1.41 ± 2.93	1.73 ± 2.61		0.04 ± 0.06	< 0.01 ± < 0.01	< 0.01 ± < 0.01
Min	0.00	< 0.01	< 0.01	< 0.01		< 0.01	< 0.01	< 0.01
Max	7.71E−06	8.26	16.32	10.126		0.26	< 0.01	< 0.01

Normally, drinking groundwater in areas with low heavy metal/metalloid contamination (lower than the standard) does not pose any health risk. For example, an assessment of the health risk from heavy metal contamination in drinking water in northern Pakistan revealed that all HQ values were lower than 1 because the heavy metal concentrations in groundwater used as drinking water were lower than the standard^54^. However, this research found that both the HQ and HI at 10 out of the 25 (40%) well stations (3, 7–9, 12–14, 19, 20 and 24) were greater than the standard, even though some well stations had Pb and/or As concentrations lower than the standards. This indicates that the risk assessment calculation also considers the intake rate (water consumption per day), which can cause a high HQ value if the daily drinking rate is high, as it was at this study site (4.21 ± 2.73 L/day).

As a result, this study revealed that even low concentrations of heavy metals in groundwater can cause adverse health effects in humans with a high water intake rate, and many types of heavy metals present a high HI risk^10,54^.

### Biomarkers

#### Urine

**Figure 2:**
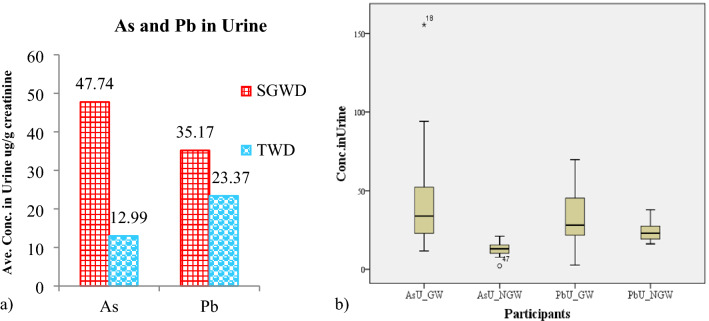
a) Comparison of urine Pb and As levels between the SGWC and TWC groups, b) Boxplot of As and Pb concentration in urine of SGWC and TWC.

The urine analysis revealed that 50% and 24.1% of the individuals in the SGWC group had higher As and Pb levels, respectively, in their urine than the standard. The standard for As in urine is 35 µg/L, and that for Pb in urine is 50 µg/g creatinine (from ACGIH, The Association Advancing Occupational And Environmental Health). On the other hand, none of the individuals in the TWC group had As or Pb levels in their urine above the standard. The average As concentration in the urine of the SGWC participants was 47.74 ± 40.76 µg/L, ranging from 3.30 to 166.60 µg/L, while the concentration in TWC participants was 12.99 ± 6.76 µg/L, ranging from 1.60 to 28.80 µg/L. The average concentration of Pb in the urine of the SGWC participants was 35.17 ± 19.84 µg/g creatinine, ranging from 1.87 to 79.73 µg/g creatinine, while the average was 23.37 ± 9.14 µg/g creatinine, ranging from 6.42 to 38.42 µg/g creatinine for the TWC participants (Fig. 2). The comparison of As and Pb in urine between SGWC and TWC, the results of Mann–Whitney U-test found As concentration in Urine of SGWC is significant different between TWC (p < 0.05) while Pb in urine is not significant different. Previous studies have used urine as a human biomarker for recent heavy metal/metalloid exposure, including studies in Egyptian residents^56^ and occupationally exposed populations in northern Japan^44^, Spain^55^ and the U.S.A.^17^. They concluded that urine is a good biomarker for heavy metal/metalloid exposure.

#### Hair and nails

**Figure 3:**
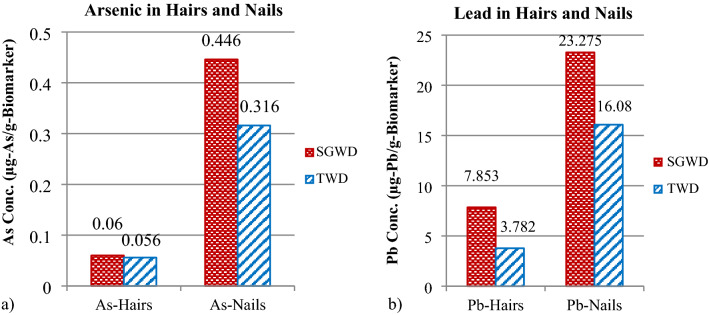
Comparison of the (**a**) As and (**b**) Pb levels in the hairs and finger nails from the SGWC and TWC groups.

The fingernail samples had higher levels of As and Pb than the hair samples (Table 3). The average As and Pb concentrations in hair were 0.06 and 7.85 µg/g hair, respectively, of the SGWC group and only 0.05 and 3.78 µg/g hair, respectively, in the TWC group. In fingernails, the average As and Pb concentrations were 0.45 and 23.28 µg/g, respectively, in the SGWC group and 0.32 and 16.08 µg/g, respectively, in the TWC group (Fig. 3).

**Table 3:** As and Pb levels in hair and fingernails.

Groundwater for consumption						Tap water for consumption								
St	Avg. As		Avg. Pb	Avg. As	Avg. Pb	St	Avg. As		Avg. Pb		Avg. As		Avg. Pb	
	(µg/g hair)		(µg/g hair)	(µg/g nails)	(µg/g nails)		(µg/g hair)		(µg/g hair)		(µg/g nails)		(µg/g nails)	
well 3	0.02		2.49	0.28	13.00	well 1	0.06		4.42		0.34		19.59	
well 4	0.11		5.87	0.89	22.03	well 2	0.05		3.45		0.41		19.07	
well 7	0.04		8.90	0.50	28.80	well 4	0.06		2.52		0.09		4.66	
well 8	0.04		7.36	0.38	21.11	well 5	0.09		3.21		0.21		6.91	
well 9	0.09		3.49	0.69	42.91	well 6	0.01		2.01		0.21		11.79	
well 11	0.04		8.35	0.46	34.81	well 9	0.15		1.73		0.04		1.81	
well 12	0.12		3.07	0.38	17.19	well 10	0.02		4.61		0.17		9.68	
well 13	0.06		4.42	0.55	28.18	well 11	0.08		1.25		0.06		2.36	
well 14	0.03		2.74	0.68	32.43	well 17	0.08		9.53		0.29		11.56	
well 15	0.07		7.37	0.40	17.22	well 18	0.05		1.45		0.08		3.64	
well 16	0.15		3.32	0.23	14.43	well 19	0.06		2.51		0.27		12.79	
well 18	0.01		10. 90	0.09	4.52	well 20	0.10		3.59		1.22		71.88	
well 19	0.03		19.13	0.70	25.11	well 21	0.04		2.47		0.40		12.82	
well 20	0.08		15.68	0.23	0.74	well 22	0.06		4.46		0.18		9.55	
well 21	0.01		44.13	0.54	27.82	well 23	0.05		4.00		0.86		54.34	
well 22	0.07		5.39	0.66	27.43									
well 23	0.06		5.39	0.33	15.84									
well 24	0.02		18.15	0.17	12.94									
Avg	0.06		7.85	0.45	23.28		0.05		3.78		0.32		16.08	
SD	0.08		9.20	0.33	20.76		0.05		4.27		0.36		21.70	
Median	0.04		4.78	0.35	18.35		0.05		3.04		0.24		10.63	
Min	0.01		0.80	0.03	1.19		0.01		0.65		0.04		1.56	
Max	0.40		45.70	1.60	118.94		0.20		28.69		1.93		111.42

Since there is currently no standard for As and Pb levels in hair or fingernails, the concentrations of As and Pb in these biomarkers were compared with the medians in all 100 local people. The Pb and As concentrations in both biomarkers in the SGWC group were regularly greater than the medians in all 100 participants and higher than those in the TWC group. For the hair samples, 27 (46.6%) and 38 (65.5%) individuals in the SGWC group had higher As and Pb concentrations, respectively, than the medians, compared to 22 (52.4%) and 12 (28.6%) individuals, respectively, in the TWC group. For the fingernail samples, 36 (62.1%) and 37 (63.8%) individuals in the SGWC group had higher As and Pb concentrations, respectively, than the medians, compared to 14 (33.3%) and 13 (31.0%) individuals, respectively, in the TWC group. The comparison of As and Pb in both hair and fingernails, the results of Mann–Whitney U-test found that they were not significantly different between SGWC and TWC.

As mentioned, urine is a good biomarker of recent (such as daily) exposure to As and Pb, while fingernails appear to be a better biomarker of long-term exposure to As and Pb than hair or urine. Furthermore, not only groundwater consumption but also other concomitant factors, such as smoking and alcohol consumption, can affect heavy metal/metalloid accumulation in the human body^57^. Therefore, binary logistic regression was applied to determine the influential concomitant factors in this study.

### Correlations between biomarkers and groundwater Pb and As concentrations

Kendall and Spearman correlations explained that the levels of As and Pb in the SGWC group hair and fingernails were not highly associated with their concentrations in the consumed groundwater (p > 0.05).

**Figure 4:**
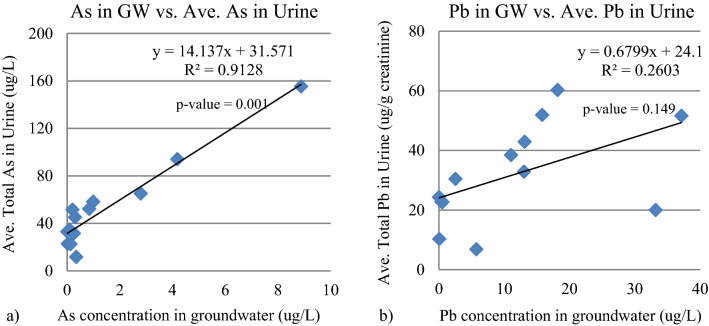
Linear relationship between the (**a**) As and (**b**) Pb levels in the urine of the SGWC group and in the groundwater (GW).

On the other hand, the Pb and As levels in urine were significantly correlated with those in the groundwater (R^2^ of 0.9128) for the SGWC group (Fig. 4). The absence of a correlation between the groundwater levels of As and Pb and their hair and nail contents was a result of the fact that hair and fingernails are not the main routes of daily excretion, whereas urine is. Rather, hair and fingernails represent long-term exposure, where several months are required for accumulation in the human body before excretion via hair and nail growth. Therefore, hair and fingernails are more subject to the effects of other concomitant factors than urine.

### Binary logistic regression

Binary logistic regression was performed to determine the significant (likely) concomitant factors affecting the levels of As and Pb in the three biomarkers. A separate cutoff point for each particular biomarker was used to categorize the data for each biomarker into two sets. Urine concentrations were separated on the basis of whether the concentration of As and Pb was higher or lower than the respective standard, whereas the hair and fingernail concentrations were separated on the basis of whether the respective As or Pb concentration was higher or lower than the median for the 100 participants. The dependent variables were the levels of As and Pb in each biomarker for all 100 participants. The independent variables were the predicted factors, such as sociodemographic, personal, exposure and environmental factors. Before performing the binary logistic regression, chi square (*X*^2^) was applied to select concomitant factors from all the factors.

#### Hair and fingernails

The *X*^2^ results showed that the significant concomitant factors affecting the As levels in hair were underlying diseases and the use of fertilizers, while for Pb, they were the consumed water source, cooking water source, use of pesticides and the use of personal protective equipment (PPE). For the fingernails, the significant concomitant factors affecting the As levels were the consumed water source, bath water source, washing water source and alcohol consumption, while for Pb, they were the consumed water source, bath water source and alcohol consumption.

The factors found using *X*^2^ analysis to be significantly associated with the Pb or As levels in the biomarkers were included in the binary logistic regression analysis to ascertain the OR that each particular factor was a possible concomitant factor, depending upon the independent variable values.

All the significant concomitant factors determined from the *X*^2^ results were used in the binary logistic regression, where probability Y and independent factor X were explored, and the results are summarized in the Supplementary Information (Table S3). Some previous studies reported heavy metals in pesticides and that the contaminated soil originated from the use of such agricultural chemicals^58^. Likewise, in Bangladesh, sex was found to be a significant concomitant factor for levels of As in nail and hair samples related to groundwater with high As levels^59^, while sex and smoking habits were strongly correlated with the heavy metal concentrations in both of these biomarkers in an Egyptian population^56^.

From the binary logistic regression results, probability Eqs. (3)–(8) were extracted from these quantities (Supplementary Information, Table S4), where the consumption of water was the most frequently observed concomitant factor in the probability equations for hair and fingernails. Thus, consuming shallow groundwater was likely to be the major source of As and Pb exposure for humans in this study area. For example, from Eq. (5), the SGWC group had a 3.58-fold higher probability risk from the As level in their nails derived from groundwater consumption than did the TWC group.

#### Urine

From the *X*^2^ analysis, the significant concomitant factors affecting As in urine were the source of consumed water and the use of fertilizer, while for Pb they were the sex, consumed water source and smoking (Supplementary Information, Table S5). The binary logistic regression revealed that the concomitant factors for Pb and As were the source of consumed water (shallow groundwater), use of fertilizer, sex and smoking. Previous research has also reported the groundwater source, sex and smoking as concomitant factors affecting heavy metals (Cd, Cr, Cu, Hg, Mn, Ni and Pb)^44,55,56^ and metalloids (As)^16,17,57^ in urine. Moreover, there was no reported Cd or Hg in the groundwater samples at a study site in the dry season^10^, which agrees with the water consumption not being a significant concomitant factor for the Cd and Hg levels in urine.

### OR analysis

Similar to the binary logistic regression results, the OR presented significant risk factors related to the As and Pb levels in groundwater. The risk factor for As in urine was the source of consumed water (OR 2.63, 95% CI 1.95–3.54), while for Pb, its was the consumed water source (OR 7.02, 95% CI 1.92–25.65) and gender (OR 2.78, 95% CI 1.03–7.48). For hair, the OR results revealed that the significant risk factor for As was smoking, while for Pb, it was the consumed water source.

In addition, the OR analysis revealed that the significant risk factors related to As in fingernails were the bath water sources (OR 4.67, 95% CI 1.59–13.88) and washing water sources (OR 4.32, 95% CI 1.46–12.82), while for Pb is the bath water sources (OR 4.36, 95% CI 1.55–12.21). Interestingly, the significant risk factors revealed by the OR analysis strongly supported the binary logistic regression results, with groundwater using being the main factor (Supplementary Information, Table S6). Similarly, some previous studies have reported groundwater consumption as one of the important concomitant factors for As in hair and nails^60–63^.

The results from both the binary logistic regression and OR analyses showed that the consumed water source, especially when groundwater, was the main risk factor. Both bathing and washing with groundwater were impacted by concomitant factors related to As and Pb accumulation in the human body.

Furthermore, previous studies have confirmed the concomitant factors found to affect heavy metals/metalloids found in hair and nails. In Egypt, sex and smoking habits were concomitant factors for toxic heavy metals in human blood, urine, hair and nails^56^, while in Bangladesh, sex had a significant impact on the As mass fraction in biomarkers^59^, where the As content in hair and nails was up to two-fold higher in females than in males. A significant difference in the As content in both male and female hair was also associated with the use of As-enriched groundwater. Likewise, in Pakistan, the amount of As in smokers’ hair (0.94 ± 0.21 µg As/g) was substantially higher than that in nonsmokers’ hair (0.43 ± 0.18 µg As/g)^64^. Furthermore, other researchers reported that the water source was one of the main concomitant factors for heavy metals^17,60–63^, which is similar to this study. Because of heavy metal exposure via the oral route, not only drinking water but also dietary consumption should be considered. Several studies have reported that food, in addition to drinking water, is associated with heavy metals in the human body^18,20,44,56,61^. For this agricultural area that contained heavy metals in soil and groundwater, the plants or crops, such as rice, tubers, and vegetables, grown in this area may pose a risk of heavy metal exposure.

## Conclusions

The health risk assessment of As and Pb revealed higher risk for the SGWC residents than for the TWC residents. In the same way, the As and Pb levels in all three biomarkers studied (hair, fingernails, and urine) were greater in the SGWC residents than in the TWC residents. Finally, the OR and binary logistic regression analyses revealed similar concomitant factors affecting Pb and As in these three biomarkers, with groundwater consumption being the most common. Therefore, this study suggested that groundwater should be used for other purposes and not as a consumed water supply or for bathing and washing. Even though As and Pb were found at low concentrations in the groundwater, they had a long-term exposure effect on human health. Furthermore, some of the shallow groundwater wells contained levels exceeding the safety standard, and such water from these agricultural areas should not be used for direct consumption without treatment. Thus, the Thai government should provide alternatives sources of consumable water to these residents, or the groundwater should be filtered properly before use. This study can serve as a guideline and database for disease prevention. This health risk assessment provides a prediction of adverse effects to prevent future harm. If no action is taken until disease occurs, it will be more difficult to help people; prevention is better than treatment. Human biomarkers (urine, hair, nails) provide evidence indicating that local people were exposed daily to heavy metals that accumulated over a long period. They may suffer adverse health effects in the future if groundwater consumption continues.

## Supplementary Information

Supplementary Information.
