# Supplementary material for: Human biomarkers associated with low concentrations of arsenic (As) and lead (Pb) in groundwater in agricultural areas of Thailand
Source: Sci Rep. 2021 Jul 6;11:13896. doi: 10.1038/s41598-021-93337-y (PMC8260595; doi:10.1038/s41598-021-93337-y)
Supplement: Supplementary file 1 — Supplementary Information. [file 41598_2021_93337_MOESM1_ESM.docx]

**Supplementary Information**

**Human Biomarkers Associated with Low Concentrations of**

**Arsenic (As) and Lead (Pb) in Groundwater in Agricultural Areas of Thailand**

Pokkate Wongsasuluk^1,2,3^, Srilert Chotpantarat^2,4,5,6*^, Wattasit Siriwong^3,7^ and Mark Robson^7,8,9^

^1^International Postgraduate Programs in Environmental Management, Graduate School, Chulalongkorn University, Bangkok 10330, Thailand.

^2^Center of Excellence on Hazardous Substance Management (HSM), Chulalongkorn University, Bangkok 10330, Thailand. Tel.: (662)2188132; Fax: (662)2554967; E-mail: pokkate_wong@hotmail.com.

^3^College of Public Health Science, Chulalongkorn University, Bangkok 10330, Thailand. Tel.: (662) 2188049.

^4^Department of Geology, Faculty of Science, Chulalongkorn University, Bangkok 10330, Thailand. Tel.: (662) 2185442; Fax: (662)2185464; e-mail: csrilert@gmail.com.

^5^Research Program on Controls of Hazardous Contaminants in Raw Water Resources for Water Scarcity Resilience, Center of Excellence on Hazardous Substance Management (HSM), Chulalongkorn University, Bangkok 10330, Thailand.

^6^Research Unit Control of Emerging Micropollutants in Environment, Chulalongkorn University, Thailand

^7^Thai Fogarty ITREOH Center, Chulalongkorn University, Bangkok 10330, Thailand. Tel.: (662) 2188182; Fax: (662)2556046; e-mail: wattasit.s@chula.ac.th.

^8^New Jersey Agricultural Experiment Station, Rutgers University, New Brunswick, NJ, USA.

^9^School of Environmental and Biological Sciences, Rutgers University, New Jersey, USA. Tel.: (732)9328165; Fax: (732)9323844; E-mail: robson@aesop.rutgers.edu.

**^*^Corresponding author. Tel.**: (662)2185442; **fax**: (662)2185464; **e-mail**: csrilert@gmail.com.

Table S1. The % relative accuracy and relative error of ICP-MS measurement for each heavy metals

| Heavy Metals | %Relative Accuracy (Ave.+SD) | Range | %Relative Error |
| --- | --- | --- | --- |
| As | 99.54+3.84 | 94.41-106.1 | 3.14% |
| Cd | 98.73+4.12 | 91.76-105.4 | 3.05% |
| Pb | 102.52+4.25 | 96.21-109.6 | 3.85% |
| Hg | 98.90+4.03 | 91.46-104.28 | 3.13% |

Table S2. The % relative error of AAS measurement for each heavy metals

| Heavy Metals | %Relative Error |
| --- | --- |
| As | 5.65% |
| Cd | 2.50% |
| Pb | 4.47% |
| Hg | 2.90% |

**Table S3.** Factors showing significant association (*X*^2^ analysis) of Pb or As levels in the hair, fingernails and urine (sig. <0.2).

| **Factors** | As-H | Pb-H | As- N | Pb- N | As-U | Pb-U |
| --- | --- | --- | --- | --- | --- | --- |
| Gender | - | - | - | - | - | 0.058 |
| Weight (kg) | - | - | - | - | - | - |
| Height (cm) | - | - | - | - | - | - |
| Age (years) | - | - | - | - | - | - |
| Consuming Rate (L) | - | - | - | - | - | - |
| Consuming Source | - | 0.001 | 0.087 | 0.069 | 0.000 | 0.000 |
| Consuming Water Container | - | - | - | - | - | - |
| Consuming Water Cleaning Method | - | - | - | - | - | - |
| Bath Water Source | - | - | 0.004 | 0.005 | - | - |
| Washing Water Source | - | - | 0.015 | - | - | - |
| Cooking Water Source | - | 0.165 | - | - | - | - |
| Education | - | - | - | - | - | - |
| Occupation | - | - | - | - | - | - |
| Family Occupation | - | - | - | - | - | - |
| Family Members (persons) | - | - | - | - | - | - |
| Work Rate | - | - | - | - | - | - |
| Smoking Behavior | - | - | - | - | - | 0.098 |
| Alcohol Drinking Behavior | - | - | 0.065 | 0.168 | - | - |
| Underlying Diseases | 0.096 | - | - | - | - | - |
| Pesticide Use | - | 0.194 | - | - | - | - |
| Chemical Fertilizer Contact | 0.132 | - | - | - | 0.170 | - |
| Washing Hands Before Meals | - | - | - | - | - | - |
| Personal Protective Equipment Use | - | 0.070 | - | - | - | - |

**Table S4.** Factors related to As and Pb concentrations in hairs and fingernails.

| **Independent factor** | | | **B** | | **Exp(B)** | | **95%CI for EXP(B)** | | | |  |
| --- | --- | --- | --- | --- | --- | --- | --- | --- | --- | --- | --- |
|  |  |  |  |  |  |  | Lower | | Upper | |  |
| Underlying Disease |  | |  | | 2.169 | | 0.924 | | 5.094 | |  |
| Fertilizer Using |  |  |  | | 2.436 | | 0.863 | | 6.876 | |  |
| Constant |  |  | -0.756 | | 0.470 | |  | |  | |  |
| Equation (3) As-Hair: | Y = -0.756 + 2.169X_12_ + 2.436X_14_  where Y = Probability that As concentration in hair higher than median. | | | | | | | | | |  |
| **Independent Factors** | | | | **B** | | **Exp(B)** | | **95%CI for EXP(B)** | | | |
|  |  |  |  |  |  |  |  | Lower | | Upper | |
| Consuming Water Source | |  | |  | | 1.331 | | 0.455 | | 3.895 | |
| Cooking Water Source | |  |  |  | | 1.672 | | 0.495 | | 5.655 | |
| Pesticides Using | |  |  |  | | 1.108 | | 0.447 | | 2.746 | |
| PPE Using | |  |  |  | | 0.802 | | 0.347 | | 1.851 | |
| Constant | |  |  | -0.515 | | 0.598 | |  | |  | |
| Equation (4) Pb-Hair: | | Y = -0.515 + 1.331X_1_ + 1.672X_17_ + 1.108X_13_ + 0.802X_18_  where Y = Probability that Pb concentration in hair higher than median. | | | | | | | | | |
| **Independent Factors** | | | | **B** | | **Exp(B)** | | **95%CI for EXP(B)** | | | |
|  |  |  |  |  |  |  |  | Lower | | Upper | |
| Consuming Water Source | |  | |  | | 3.578 | | 1.278 | | 10.013 | |
| Alcohol Drinking | |  |  |  | | 2.539 | | 0.921 | | 7.001 | |
| Bath Water Source | |  |  |  | | 17.327 | | 1.480 | | 202.814 | |
| Washing Water Source | |  |  |  | | 2.140 | | 0.205 | | 22.342 | |
| Constant | |  |  | -3.325 | | 0.036 | |  | |  | |
| Equation (5) As-Nail: | | Y = -3.325 + 3.578X_1_ + 2.539X_10_ + 17.327 X_2_ + 2.140X_3_  where Y = Probability that As concentration in nail higher than median. | | | | | | | | | |
| **Independent Factors** | | | | **B** | | **Exp(B)** | | **95%CI for EXP(B)** | | | |
|  |  |  |  |  |  |  |  | Lower | | Upper | |
| Consuming Water Source | |  | |  | | 1.113 | | 0.388 | | 3.192 | |
| Bath Water Source | |  |  |  | | 4.498 | | 1.233 | | 16.416 | |
| Alcohol Drinking | |  |  |  | | 1.855 | | 0.700 | | 4.914 | |
| Constant | |  |  | -1.322 | | 0.267 | |  | |  | |
| Equation (6) Pb-Nail: | | Y = -1.322+ 1.113 X_1_ + 4.498X_2_ + 1.855X_10_  where Y = Probability that Pb concentration in nails higher than median. | | | | | | | | | |

*(X_1_= Consuming Source, X_2=_ Bath water source, X_3_=Washing water Source, X_4_=Consuming Rate, X_5_=Gender, X_6_=Height, X_7=_ Education, X_8_=Occupation, X_9_=Smoking, X_10_=Alcohol Drinking, X_11_=Working Hour per day, X_12=_Underlying Diseases, X_13_=Pesticides Using, X_14_=Fertilizer Using, X15=Age, X16=Weight, X17=Cooking Water Source, X18=PPE Using)

**Table S5.** Factors related with concentration of As and Pb in urine.

| **Independent factor** | | **B** | **Exp(B)** | **95%CI for EXP(B)** | |
| --- | --- | --- | --- | --- | --- |
|  |  |  |  | Lower | Upper |
| Consuming Water Source |  |  | 3.704 | 1.258 | 10.902 |
| Fertilizer Using |  |  | 2.372 | 0.616 | 9.131 |
| Constant |  | -2.537 | 0.079 |  |  |
| Equation (7) As-Urine: | Y = -2.537 + 3.704X_1_ + 2.372X_14_  where Y = Probability that As concentration in urine higher than standard. | | | | |
| **Independent Factors** | | **B** | **Exp(B)** | **95%CI for EXP(B)** | |
|  |  |  |  | Lower | Upper |
| Consuming Water Source |  |  | 6.275 | 1.693 | 23.254 |
| Gender |  |  | 0.553 | 0.105 | 2.917 |
| Smoking |  |  | 1.273 | 0.234 | 6.934 |
| Constant |  | -2.220 |  |  |  |
| Equation (8) Pb-Urine: | Y = -2.220 + 6.275X_1_ + 0.553X_5_ + 1.273X_9_  where Y = Probability that Pb concentration in urine higher than standard. | | | | |

*(X_1_= Consuming Source, X_2=_ Bath water source, X_3_=Washing water Source, X_4_=Consuming Rate, X_5_=Gender, X_6_=Height, X_7=_ Education, X_8_=Occupation, X_9_=Smoking, X_10_=Alcohol Drinking, X_11_=Working Hour per day, X_12=_Underlying Diseases, X_13_=Pesticides Using, X_14_=Fertilizer Using, X15=Age, X16=Weight, X17=Cooking Water Source, X18=PPE Using)

**Table S6.** OR analysis of the risk factors related with As and Pb in hair, fingernails and urine.

| **Heavy Metals** | **Hair-Risk Factors** | |  |  | | **95%CI** | | |  |
| --- | --- | --- | --- | --- | --- | --- | --- | --- | --- |
|  |  |  |  | **OR** | | **Lower** | **Upper** | |  |
| As-H | Underlying Disease | |  | 2.135 | | 0.922 | 4.944 | |  |
|  | Use of Fertilizers | |  | 2.388 | | 0.861 | 6.618 | |  |
|  |  | |  |  | |  |  | |  |
| Pb-H | Consuming Water Source | |  | 3.857 | | 1.670 | 8.911 | | Sig. |
|  | Cooking Water Source | |  | 2.144 | | 0.842 | 5.459 | |  |
|  | Use of Pesticides | |  | 1.941 | | 0.818 | 4.607 | |  |
|  | Use of PPE | |  | 0.442 | | 0.198 | 0.987 | |  |
| **Heavy Metals** | **Nail-Risk Factors** | |  |  | | **95%CI** | | |  |
|  |  |  |  | **OR** | | **Lower** | **Upper** | |  |
| As-N | Consuming Water Source | |  | 2.17 | | 0.966 | 4.874 | |  |
|  | Alcohol Drinking | |  | 2.523 | | 0.989 | 6.441 | |  |
|  | Bath Water Source | |  | 4.694 | | 1.588 | 13.877 | | sig |
|  | Washing Water Source | |  | 4.32 | | 1.456 | 12.818 | | sig |
| Pb-N | Consuming Water Source | |  | 2.072 | | 0.929 | 4.626 | |  |
|  | Bath Water Source | |  | 4.355 | | 1.553 | 12.21 | | Sig. |
|  | Alcohol Drinking | |  | 2.032 | | 0.798 | 5.171 | |  |
| **Heavy Metals** | | **Urine-Risk Factors** |  |  | **95%CI** | | | |  |
|  |  |  |  | **OR** | **Lower** | | | **Upper** |  |
| As-U | | Consuming Water Source |  | 2.63 | 1.954 | | | 3.539 | Sig. |
|  | | Use of Fertilizers |  | 2.728 | 0.734 | | | 10.148 |  |
|  | |  |  |  |  | | |  |  |
| Pb-U | | Consuming Water Source |  | 7.018 | 1.92 | | | 25.654 | Sig. |
|  | | Gender |  | 2.778 | 1.031 | | | 7.482 | Sig. |
|  | | Smoking |  | 2.484 | 0.909 | | | 6.791 |  |

*Water source = Tap water is reference and groundwater is risk factor.
